# Probing the Reaction Mechanisms of 3,5-Difluoro-2,4,6-Trinitroanisole (DFTNAN) through a Comparative Study with Trinitroanisole (TNAN)

**DOI:** 10.3390/ma15072568

**Published:** 2022-03-31

**Authors:** Qingjie Jiao, Tianqi Li, Yapeng Ou, Suming Jing, Fang Wang

**Affiliations:** 1 State Key Laboratory of Explosion Science and Technology, Beijing Institute of Technology, Beijing 100081, China; jqj@bit.edu.cn (Q.J.); 3220190133@bit.edu.cn (T.L.); 2 School of Environment and Safety Engineering, North University of China, Taiyuan 030051, China; 20160075@nuc.edu.cn; 3 Science and Technology on Aerospace Chemical Power Laboratory, Hubei Institute of Aerospace Chemotechnology, Xiangyang 441003, China

**Keywords:** melt-cast explosive, thermal decomposition, fluorinated energetic materials, transferred trigger bond

## Abstract

To probe the thermal decomposition mechanisms of a novel fluorinated low-melting-point explosive 3,5-difluoro-2,4,6-trinitroanisole (DFTNAN), a comparative study with trinitroanisole (TNAN) was performed under different heating conditions. The thermal decomposition processes and initial reactions were monitored by DSC-TG-FTIR-MS and T-jump-PyGC-MS coupling analyses, respectively. The results show that fluorine decreased the thermal stability of the molecular structure, and the trigger bond was transferred from the ortho-nitro group of the ether to the para-nitro group. The possible reaction pathway of DFTNAN after the initial bond breakage is the rupture of the dissociative nitro group with massive heat release, which induces the ring opening of benzene. Major side reactions include the generation of polycyclic compounds and fluorine atom migration. Fluorine affects the thermal stability and changes the reaction pathway, and fluorinated products appear in the form of fluorocarbons due to the high stability of the C-F bond.

## 1. Introduction

3,5-Difluoro-2,4,6-trinitroanisole (DFTNAN) is a recently synthesized energetic material with a melting point of 82 °C [1]. It exhibits great potential to become the next generation melt-cast explosive carrier due to the ideal energy performances (with a density of 1.81 g/cm^3^ and a detonation velocity of 8540 m/s) and insensitivities. Melt-cast explosives are one of the most popular charges of warheads, especially in certain large weapon systems, such as torpedoes and ballistic missiles, due to the easy and continuous manufacturability [2].

However, the disadvantages, such as poor stability and energetic performances of conventional melt-cast explosive carriers, such as 2,4,6-trinitrotoluene (TNT) [3], urged the development of novel low-melting-point energetic materials. In past decades, many N-heterocyclic compounds, such as DNBP (4,4′-dinitro-3,3′-bifurazan), MTNP (1-methyl-3,4,5-trinitropyrazole), TNAZ (1,3,3-trinitroazetidine) and derivatives of 1,2,5-oxadiazole-2-oxide (furoxan) were studied as potential melt-cast explosive carriers due to their high nitrogen content and moderate sensitivity [4,5].

A recent breakthrough was the introduction of fluorine atom or fluorine-containing groups, such as NF_2_ and SF_5_, into molecules [6,7]. Fluorine is the most electronegative element known, which gives fluorinated energetic materials high energy and density, excellent stability and strong oxidizing capacity [8,9]. In addition to the remarkable performances of fluorinated energetic materials, their interactions with metallic fuels also draw attention [10,11]. Theoretically, fluorination of per mole metallic fuel can produce more energy than oxidation (Δ*_f_H*AlF_3_ = −1510 kJ/mol, Δ*_f_H*AlO_1.5_ = −838 kJ/mol); and metal fluorides typically have much lower boiling points than their respective metal oxides (*T*_b.p._ (BF_3_) = −100 °C, *T*_b.p._(B_2_O_3_) = 1860 °C).

These characteristics make fluorinated energetic materials a positive application prospect. However, mainstream research suggests that the decomposition of fluorinated energetic materials cannot directly produce free fluorine due to the high stability of C-F bond [12]. Instead, fluorine-containing free radicals generated from decomposition are the most reactive intermediates, which actually associate with metallic fuels to produce heat [13,14]. Therefore, the decomposition pathway of fluorinated energetic materials and their reactive products are of significance to subsequent interactions and energy release for energetic composites.

The current work attempts to probe the possible reaction mechanisms of DFTNAN during thermal decomposition through a comparative study with trinitroanisole (TNAN) by in situ monitoring of the gaseous products from non-isothermal decomposition and flash pyrolysis. Non-isothermal differential scanning calorimetry–thermogravimetry–mass–spectrometry–Fourier-transform infrared spectroscopy coupling analysis (DSC-TG-MS-FTIR) and T–jump–pyrolysis gas chromatograph–mass spectrometry coupling analysis (T–jump–PyGC–MS) are used as analytical techniques.

## 2. Experimental Section

### 2.1. Materials

**Figure 1:**
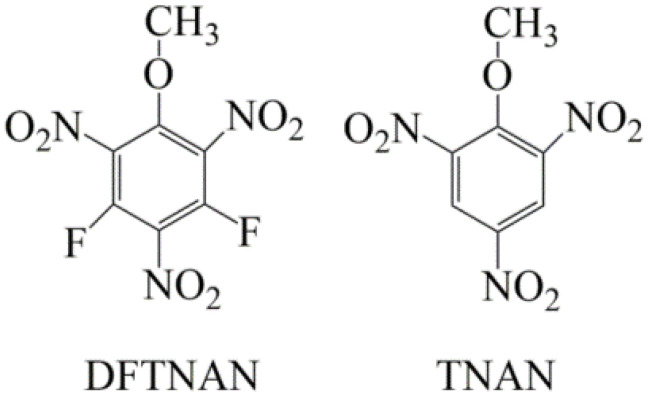
The molecular structures of DFTNAN and TNAN.

3,5-Difluoro-2,4,6-trinitroanisole (DFTNAN) (97.6%, HPLC) and 2,4,6-trinitroanisole (TNAN) (99.0%, HPLC) were provided by North University of China (Taiyuan, China), and their molecular structures are shown in Figure 1.

### 2.2. Instruments and Characterizations

Non-isothermal differential scanning calorimetry–thermogravimetry–mass spectrometry–Fourier-transform infrared spectroscopy coupling analysis (DSC-TG-MS-FTIR) was performed using Netzsch STA449C (Selb, Germany), Netzsch-QMS403C and Bruker VERTEX 70 (Rosenheim, Germany). A total of 2.0 mg of sample was heated from 30 to 400 °C at a heating rate of 10 °C/min under an Ar atmosphere flow of 50 mL/min.

T-jump pyrolysis was performed using a CDS Analytical (Note: CDS Analytical is the name of manufacture) Pyroprobe 5150 (Oxford PA, USA) coupling with a Thermal Fisher ISQ series gas chromatograph (GC) and Py-mass spectrometry. Flash pyrolysis was performed under a heating rate of 1500 °C/min, from ambient temperature to 530 °C, and the isothermal pyrolysis time was set as 5 s.

## 3. Results

### 3.1. Thermal Decomposition Process

**Figure 2:**
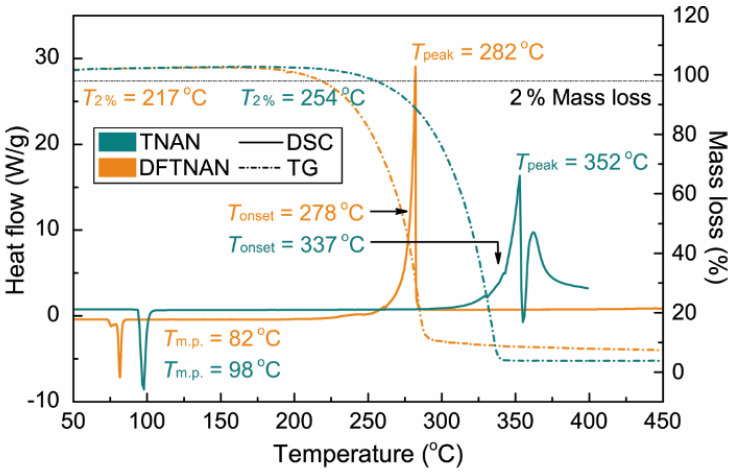
DSC and TG curves of the decomposition processes of DFTNAN and TNAN.

The DSC-TG curves of DFTNAN and TNAN at a heating rate of 10 °C/min are shown in Figure 2. An endothermic peak can be observed at 82 °C on a DSC curve of DFTNAN without mass loss. This endothermic peak is ascribed to the melting process of DFTNAN, which is consistent with previous research [15]. The mass loss of DFTNAN appears from 217 to 285 °C on the TG curve; however, the decomposition starts from 278 °C (*T*_onset_) with an apparent exothermic peak at 282 °C (*T*_peak_).

Before that, a slow volatilization can be recognized from 210 °C with an overlapped peak at 255 °C on the DTG curve (Figure S1). The melting point of TNAN is about 98 °C. Interestingly, there are two separated exothermic peaks at 352 and 362 °C, respectively on the DSC curve of TNAN. TNAN undergoes decomposition in this temperature region, which starts from 337 °C. This could be caused by the different decomposition processes of gasified and condensed TNAN since it has a very low boiling point.

It is unexpected that the thermal decomposition temperatures of DFTNAN is 55 °C lower than that of TNAN since fluorinated compounds usually show considerably higher thermal stability and lower sensitivities due to the C-F bond [16,17]. However, in Reichel’s work [18], fluorination leads to an energetically destabilizing effect of methyl nitrate and its fluorinated derivatives, which is caused by the shorter C-O and N-O bonds in molecular structures.

### 3.2. Time-Resolved Decomposition Products

For a clear understanding of the reaction mechanisms of DFTNAN and the effect of fluorine on the thermal stability, the gaseous decomposition products of DFTNAN and TNAN are in situ monitored. It can be observed from the 3D FTIR images (Figure S2) and time-resolved MS curves (Figure S3) of the gaseous products that the thermal decomposition of DFTNAN is a three-stage process. The first and second stages occur before 300 °C corresponding to the *T*_onset_ and *T*_peak_, respectively. It can be speculated that evolved gaseous products of the third stage are generated by the interaction of intermediates since the thermal decomposition process finished after 300 °C on the DSC curve.

**Figure 3:**
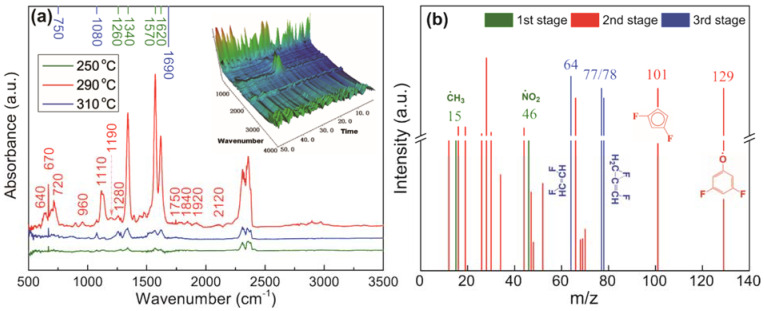
(**a**) FTIR spectra and (**b**) MS patterns of time-resolved decomposition products of DFTNAN.

Figure 3 depicts the FTIR spectra of DFTNAN at different temperatures with time-resolved 3D images and MS patterns at the corresponding stages. At the first stage, only NO_2_ and CH_3_ radicals can be observed (the m/z of Py-MS and possible assignments are listed in Table S1), which have absorption bands at 1620 and 1570 cm^−1^, and 1340 cm^−1^, respectively at FTIR spectrum [19,20]. Correspondingly, fragment ions with m/z of 46 and 15 can be detected by MS. The main decomposition process occurs at the second stage.

Except the rising peaks attributed to NO_2_ and CH_3_ ions, a very apparent peak can be observed at 1110 cm^−1^, which is attributed to the stretching vibration of C-F bond (this can also be determined by comparison of the FTIR spectra of DFTNAN and TNAN available in Figure S4) [21]. A weak band at 1280 cm^−1^ can be attributed to the phenolic hydroxyl of C_6_H_3_F_2_O with m/z of 129, generated from the disassociation of methyl from the ether group and nitro group. The broad band at 720 cm^−1^ is attributed to HCN corresponding to the fragment ion with m/z of 27, which is formed from the dissociative methyl and nitro group [22].

Weak bands at 960, 1190, 1750 and 1840 cm^−1^ indicate the generation of aldehydes and anhydrides, which are caused by the oxidation of intermediates after the breakage of the C-O bond between benzene and ether. The absorption band at 1920 cm^−1^ and fragment ion with m/z of 28 indicate the generation of CO; meanwhile, a five-membered ring with m/z of 101 (C_5_H_3_F_2_) reveals the benzene ring-opening reaction. The absorption bands at 640, 670 and 2120 cm^−1^, and the fragment ion with m/z of 26 show the generation of alkyne after the five-membered ring opens [23]. In addition, fluoroalkanes, including CH_3_F, CH_2_F_2_ and CHF_3_, can be detected by MS with m/z of 34, 52 and 70, respectively.

Products of the third stage are mainly from the secondary reactions of intermediates. A sharp peak at 1080 cm^−1^ is attributed to *v*(C-O-C) in ether, which could be caused by simple radical–radical recombination reactions from generated aldehydes and alkanes [24]. Broad bands around 750 and 1690 cm^−1^ are attributed to *β*(C-H) and *v*(C=C) in alkenes. Fragment ions with m/z of 77 and 78 are also detected, which indicate the generation of C_3_F_2_H_3_ and C_3_F_2_H_4_ from ring opening. The fluorine atom in C_3_F_2_H_4_ migrates with the increasing temperature [25] and further decomposes into C_2_H_2_F_2_ with a m/z of 64.

**Figure 4:**
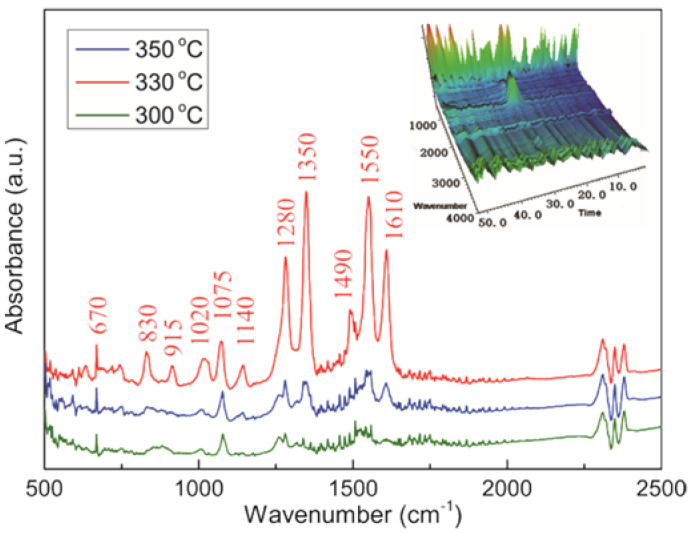
FTIR spectra of time-resolved decomposition products of TNAN.

As shown in Figure 4, NO_2_ and CH_3_ radicals can be detected in the first stage of the decomposition process of TNAN with absorption peaks at 1610, 1550 and 1350 cm^−1^ (the corresponding MS patterns can be found in Figure S5). TNAN undergoes fast decomposition with temperature increasing and generates similar products with DFTNAN [26]. The peak at 1075 cm^−1^ is attributed to *v*(C-O-C) in ether, the peak at 1280 cm^−1^ is attributed to phenolic hydroxyl, and the peak at 670 cm^-1^ is attributed to *β*(=C-H) of alkyne.

The peak at 915 cm^−1^ is attributed to *v*(C-N) in the nitro group, indicating that some nitro groups are still attached to benzene ring. The peaks at 830 and 1020 cm^−1^ are attributed to *v*(C-N) and *v*(N-H) in amine (-C-NH_2_), respectively. This suggests that some attached nitro groups are reduced to amine [27]. The peaks at 1140 and 1490 cm^−1^ are attributed to *v*(C-C) and *v*(-CH_2_-), respectively, which suggests the benzene ring-opening reaction and the generation of short-chain hydrocarbons.

### 3.3. Initial Reactions of DFTNAN and TNAN

Above thermo-analytical measurements and evolved gas analysis coupling methods avails the in situ monitoring of the gaseous products of DFTNAN and TNAN. However, the ignition and combustion processes of energetic materials usually exhibit heterogeneity and include multiple phases [13]. To fully understand the initial reactions occurring to thermal stimulus, the heating rate must approach the ignition process, and the reaction should occur at the combustion temperature. A new pulse pyrolysis equipment called the T-jump pyrolyzer was developed recently and can provide an extremely high heating rate of 20 °C/ms [28]. This technique is able to truly describe the processes of ignition and combustion.

**Figure 5:**
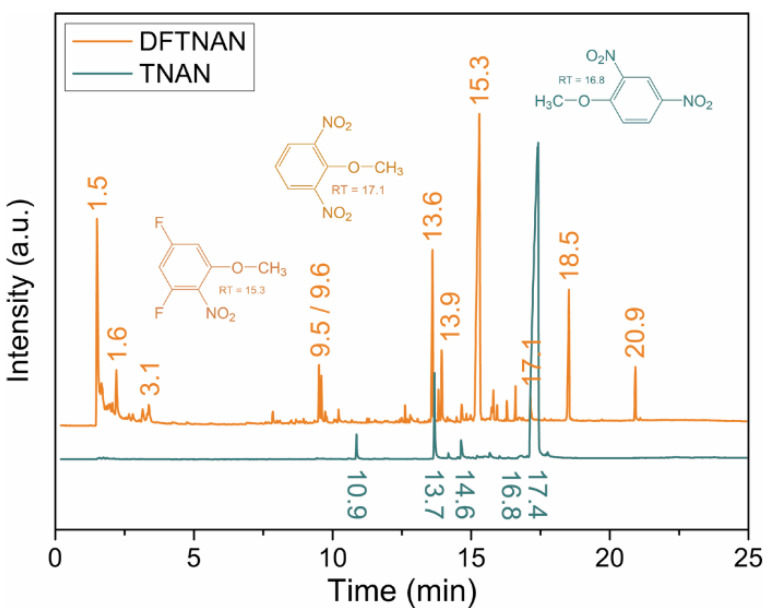
GC curves of the gaseous decomposition products of DFTNAN and TNAN after T-jump pyrolysis.

Figure 5 shows the GC curves of the gaseous decomposition products of DFTNAN and TNAN after T-jump pyrolysis (possible assignments identified by PyMS can be found in Tables S2 and S3). The most significant product of DFTNAN is 3,5-difluoro-2-nitroanisole (DFNAN) (m/z = 189) appearing at 15.3 min. Since DFTNAN has a symmetrical molecular structure, this product reveals that the trigger bond of the thermal decomposition of DFTNAN is the C-N bond of the nitro group in the para position. This initial reaction is also the primary process, which gives DFNAN an intensity of 40.84%.

Another illustrative product is 2,6-dinitroanisole (m/z = 198) with a retention time of 17.1 min, which loses the meta-nitro group. Different from non-isothermal decomposition, it is interesting that the stable C-F bond is also broken during pulse pyrolysis due to the high intensity stimulus. 3,5-Difluoro-2-nitrophenol (m/z = 175) at 14.7 min also reveals that DFTNAN undergoes disassociation of the para-nitro group and methyl from the ether group. The product at 13.6 min is C_10_H_6_N_2_O_5_. This suggests that the methyl from the ether group is cleaved after the initial reaction. These products are consistent with the results observed from FTIR. Another interesting product is CH_2_F_2_ appearing at 1.6 min. This provides direct evidence for the above speculation about F atom migration.

Some other high concentration products include five-member heterocyclic ring compounds at 9.5 and 9.6 min, and polycyclic compounds at 13.9, 18.5 and 20.9 min, which support the ring-opening and re-cyclization reactions observed during non-isothermal heating. Methylbutenol at 1.5 min is consistent with the alkene and alcohol products observed in FTIR.

After pulse heating of TNAN, the most intensive detected substance is TNAN itself appearing at 17.4 min. This suggests that TNAN has a high thermal stability and that sublimation is the primary process. However, the most significant product is 2,4-dinitroanisole (m/z = 169) at 16.8 min; it has very low intensity however. This reveals that the trigger bond of the thermal decomposition of TNAN is the C-N bond of the ortho nitro group of ether.

Another remarkable product is 4-nitrobenzaldehyde (m/z = 151), which appears at 13.7 min and is generated from the oxidation of the intermediate after ether breakage. This reaction is consistent with DFTNAN. After this reaction, aryl ether undergoes carbon rearrangement [29]. Other products include C_6_H_5_NO_2_, C_7_H_4_N_2_O_3_, C_6_H_4_N_2_O_5_ and C_7_H_6_N_2_O_4_ at 10.9, 14.6, 15.2 and 15.6 min, respectively. It is noticeable that the benzene ring has quite high thermal stability in the case of TNAN since all the gaseous products are aryl compounds.

## 4. Discussion

**Scheme 1:**
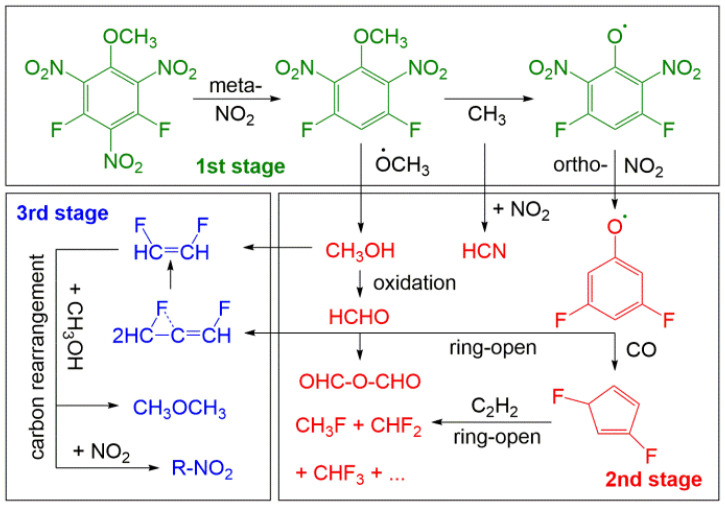
Possible decomposition reaction pathway of DFTNAN.

By identifying the thermal decomposition and pyrolysis products of DFTNAN and TNAN, we preliminarily obtain the reaction pathway of DFTNAN. As shown in Scheme 1, the para-nitro group breaks in the first stage, and the corresponding C-NO_2_ bond is the trigger bond of the whole reaction. It is different from TNAN that the first disassociated group in TNAN is the ortho-nitro group of the ether. After that, the methyl from the ether group disassociates followed by the removal of the remaining nitro groups.

In this process, the whole OCH_3_ radical may shed as a side reaction, and form methanol in the second stage. The disassociation of nitro groups generates massive heat, which prompts further decomposition, including the benzene ring opening and the generation of CO to re-cyclize a five-membered ring. Fluorine-containing cyclic compounds undergo further decomposition into short-chain fluorinated hydrocarbons. These intermediates occur as secondary reactions in the third stage, including carbon rearrangement to form simple ethers.

## 5. Conclusions

In this work, we adopted multiple thermal analysis methods to probe the reaction mechanisms of DFTNAN. By analyzing the time-resolved gaseous products during decomposition, we clarified the possible decomposition reaction pathway and major side reactions of DFTNAN and the effect of fluorine. Fluorine decreases the thermal stability of molecular bonds by transferring the trigger bond from the ortho-nitro group of the ether to the para-nitro group between two fluorine atoms. After the disassociation of nitro and the corresponding massive heat output, the ether breaks before the benzene ring-opening reaction, and the intermediates further decompose into short-chain fluorinated hydrocarbons. Finally, short-chain hydrocarbons undergo carbon rearrangement to form simple ethers to an extent. Major side reactions include the generation of polycyclic compounds and the fluorine atom migration inside the molecule.

## Data Availability

Not applicable.

## Supplementary Materials

The following supporting information can be downloaded at: https://www.mdpi.com/article/10.3390/ma15072568/s1, Figure S1: DTG curves of DFTNAN and TNAN; Figure S2: 3D FTIR spectrum of the pyrolysis gaseous products of DFTNAN and TNAN.; Figure S3: MS curves of the pyrolysis gaseous products of DFTNAN; Figure S4: FTIR spectrum of the DFTNAN and TNAN; Figure S5: MS curves of the pyrolysis gaseous products of TNAN. Table S1: The m/z of Py-MS and possible assignments; Table S2: Gaseous products of DFTNAN after T-jump pyrolysis identified by GC-MS (Retention time with corresponding molecular structures); Table S3: Gaseous products of TNAN after T-jump pyrolysis identified by GC-MS (Retention time with corresponding molecular structures).
