# Supplementary material for: Probing the Reaction Mechanisms of 3,5-Difluoro-2,4,6-Trinitroanisole (DFTNAN) through a Comparative Study with Trinitroanisole (TNAN)
Source: Materials (Basel). 2022 Mar 31;15(7):2568. doi: 10.3390/ma15072568 (PMC9000474; doi:10.3390/ma15072568)
Supplement: Supplementary file 1 [file materials-15-02568-s001.zip › materials-1657714-supplementary.pdf]

Probing the reaction mechanisms of 3,5-difluoro-2,4,6-trinitroanisole (DFTNAN) through a comparative study with trinitroanisole (TNAN)

Qingjie Jiao [a], Tianqi Li [a], Yapeng Ou [a,*], Suming Jing [b], Fang Wang [c,*]

[a] *State Key Laboratory of Explosion Science and Technology, Beijing Institute of Technology, Beijing 100081, China*

[b] *School of Environment and Safety Engineering, North University of China, Taiyuan 030051, China*

[c] *Science and Technology on Aerospace Chemical Power Laboratory, Hubei Institute of Aerospace Chemotechnology, Xiangyang 441003, Hubei, China*

# Figures

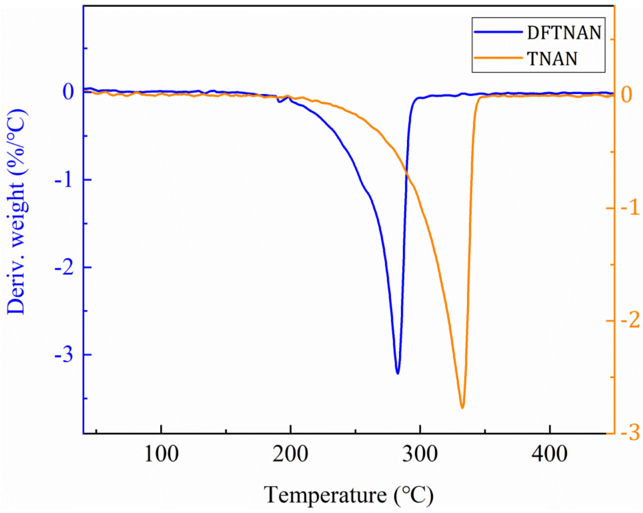


**Figure S1.** DTG curves of DFTNAN and TNAN.

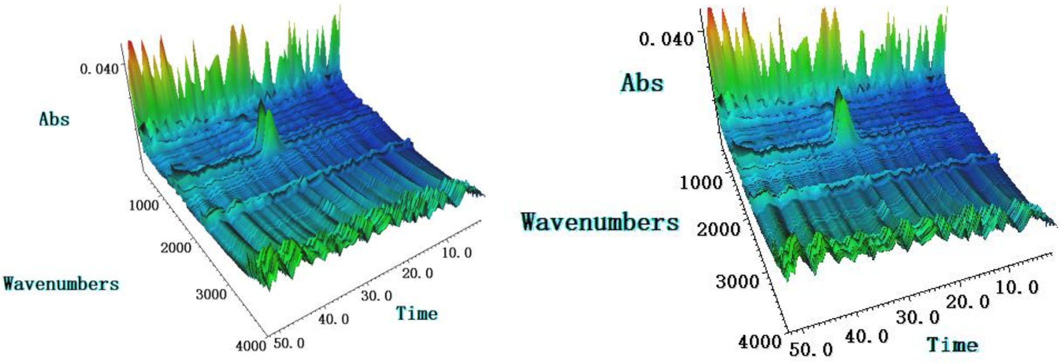


**Figure S2.** 3D FTIR spectrum of the pyrolysis gaseous products of DFTNAN and TNAN.

* Corresponding authors at: Zhongguancun South Street No. 5 Yard, Haidian District, Beijing 100081, China
E-mail addresses: ouyapeng@126.com (Y. Ou), 7520190083@bit.edu.cn (F. Wang).

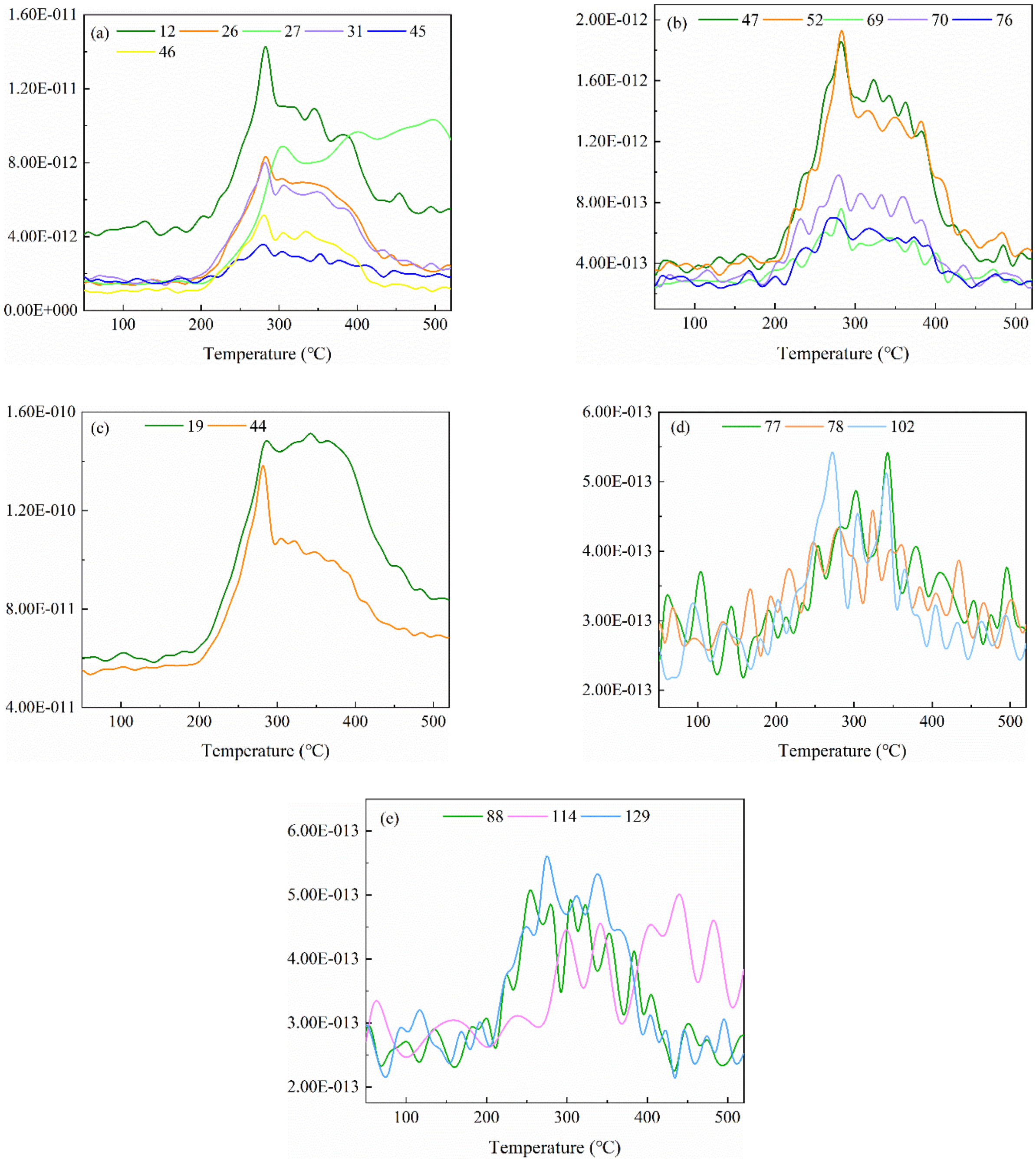


**Figure S3.** MS curves of the pyrolysis gaseous products of DFTNAN.

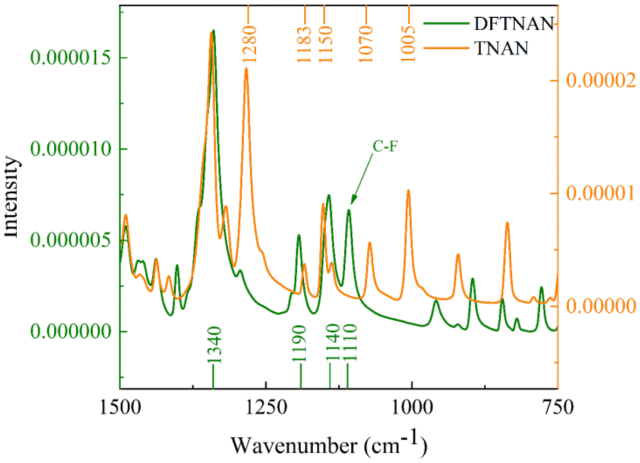


**Figure S4.** FTIR spectrum of the DFTNAN and TNAN

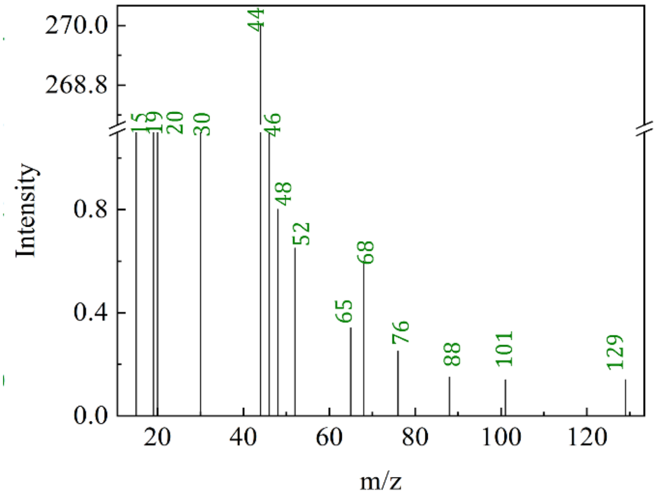


**Figure S5.** MS curves of the pyrolysis gaseous products of TNAN.

# Tables

**Table S1.** The m/z of Py-MS and possible assignments

| m/z | Possible assignments | m/z | Possible assignments |
|---|---|---|---|
| 1 | H | 44 | $CO_2$、$N_2O$ |
| 2 | $H_2$ | 45 | COOH |
| 12 | C | 46 | HCOOH,$NO_2$,$C_2FH$， $C_2H_6O$ |
| 14 | N | 47 | $C_2FH_2$ |
| 15 | $CH_3$ | 48 | $C_2FH_5$ |
| 16 | O, $CH_4$ | 50 | $CF_2$ |

| | | | |
|---|---|---|---|
| 17 | OH, $NH_3$ | 52 | $CH_2F_2$ |
| 18 | $H_2O$, $NH_4$ | 64 | $C_2H_2F_2$ |
| 19 | F | 66 | $CF_2O$ |
| 20 | HF | 68 | $NOF_2$ |
| 26 | $C_2H_2$ | 69 | $CF_3$ |
| 27 | HCN | 70 | $CHF_3$ |
| 28 | CO, $N_2$ | 76 | $C_3H_2F_2$ |
| 30 | NO | 77 | $C_3F_2H_3$ |
| 31 | CF | 78 | $C_3F_2H_4$ |
| 32 | $CH_3OH$ | 88 | $CF_4$、$C_5H_{12}O$ |
| 33 | NF | 102 | $C_5H_4F_2$ |
| 34 | $CFH_3$ | 114 | C6H4F2 |
| 38 | $F_2$ | 129 | $C_6H_3F_2O$ |

**Table S2.** Gaseous products of DFTNAN after T-jump pyrolysis identified by GC-MS (Retention time with corresponding molecular structures).

| $RT_{DFTNAN}$ | m/z | Chemical | Structure | $RT_{DFTNAN}$ | m/z | Chemical | Structure |
|---|---|---|---|---|---|---|---|
| 1.5 | 86 | $C_5H_{10}O$ | 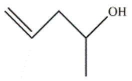 | 14.7 | 175 | $C_6H_3F_2NO_3$ | 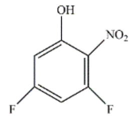 |
| 1.6 | 52 | $CH_2F_2$ | 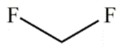 | 15.3 | 189 | $C_7H_5F_2NO_3$ | 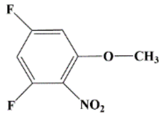 |
| 2.2 | 148 | $C_6H_{12}O_4$ | 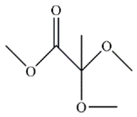 | 15.7 | 214 | $C_{11}H_6N_2O_3$ | 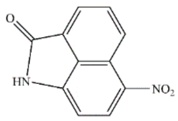 |
| 3.1 | 96 | $C_6H_5F$ | 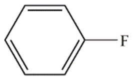 | 15.8 | 234 | $C_{10}H_6N_2O_5$ | 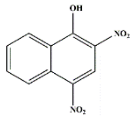 |
| 3.4 | 171 | $C_8H_{13}NO_3$ | 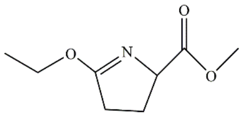 | 15.9 | 370 | $C_{20}H_{28}F_2O_4$ | 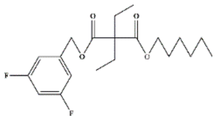 |
| 9.5 | 186 | $C_{10}H_{22}N_2O$ | 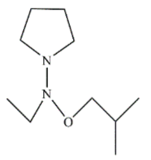 | 16.3 | 286 | $C_{14}H_{16}F_2O_4$ | 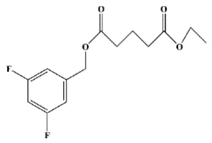 |
| 9.6 | 186 | $C_{10}H_{22}N_2O$ | 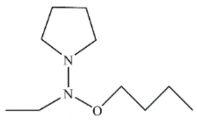 | 16.6 | 254 | $C_{16}H_{30}O_2$ | 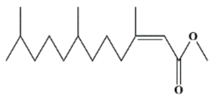 |
| 13.6 | 234 | $C_{10}H_6N_2O_5$ | 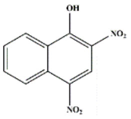 | 17.1 | 198 | $C_7H_6N_2O_5$ | 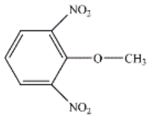 |

| | | | | | | | |
|---|---|---|---|---|---|---|---|
| 13.8 | 252 | $C_{16}H_{16}N_2O$ | 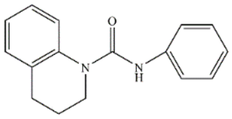 | 18.5 | 185 | $C_8H_{15}N_3O_2$ | 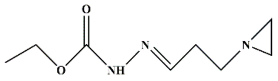 |
| 13.9 | 200 | $C_{12}H_{12}N_2O$ | 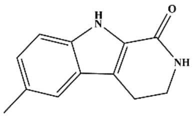 | 20.9 | 164 | $C_{12}H_{20}$ | 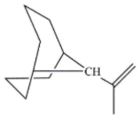 |

**Table S3.** Gaseous products of TNAN after T-jump pyrolysis identified by GC-MS (Retention time with corresponding molecular structures).

| $RT_{TNAN}$ | m/z | Chemical | Structure |
|---|---|---|---|
| 10.9 | 123 | $C_6H_5NO_2$ | 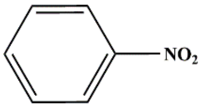 |
| 13.7 | 151 | $C_7H_5NO_3$ | 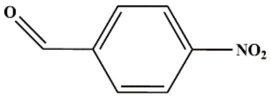 |
| 14.6 | 164 | $C_7H_4N_2O_3$ | 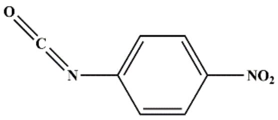 |
| 15.2 | 184 | $C_6H_4N_2O_5$ | 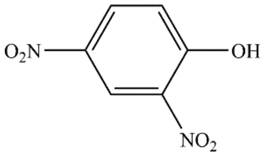 |
| 15.7 | 224 | $C_9H_8N_2O_5$ | 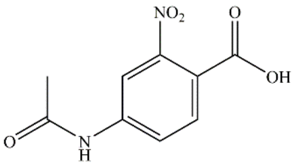 |
| 16.8 | 169 | $C_7H_7NO_4$ | 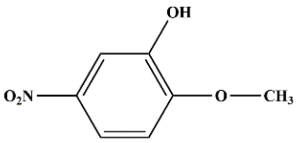 |
| 17.4 | 243 | $C_7H_5N_3O_7$ | 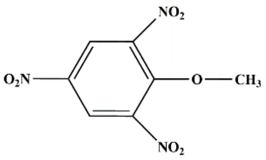 |
